# Surface Plasmon Effect Dominated High-Performance Triboelectric Nanogenerator for Traditional Chinese Medicine Acupuncture

**DOI:** 10.34133/2022/9765634

**Published:** 2022-10-07

**Authors:** Xin Chen, Fayang Wang, Yanjun Zhao, Pengfan Wu, Lingxiao Gao, Chun Ouyang, Ya Yang, Xiaojing Mu

**Affiliations:** ^1^Key Laboratory of Optoelectronic Technology & Systems Ministry of Education, International R & D center of Micro-nano Systems and New Materials Technology, Chongqing University, Chongqing 400044, China; ^2^Institute of Materials, Ecole Polytechnique Fédérale de Lausanne (EPFL), Lausanne 1015, Switzerland; ^3^School of Mechanical Engineering, Hebei University of Technology, Tianjin 300401, China; ^4^Hospital of Chongqing University, Chongqing 400044, China; ^5^CAS Center for Excellence in Nanoscience, Beijing Key Laboratory of Micro-Nano Energy and Sensor, Beijing Institute of Nanoenergy and Nanosystems, Chinese Academy of Sciences, Beijing 101400, China

## Abstract

Available, effectively converting low-frequency vibration into available electricity, triboelectric nanogenerator (TENG) is always research hot nowadays. However, the enhancing effect of the existing methods for the output have all sorts of drawbacks, i.e., low efficiency and unstable, and its practical applications still need to be further explored. Here, leveraging core-shell nanoparticles Ag@SiO_2_ doping into tribo-materials generates the surface plasmon effect to boost the output performance of the TENG. On one hand, the shell alleviated the seepage effect from conventional nanoparticles; on the other hand, the surface plasmon effect enabled the core-shell nanoparticles to further boost the output performance of TENG. We circumvent the limitations and present a TENG whose output power density can be up to 4.375 mW/cm^2^. Points is that this article novelty investigate the high-performance TENG applicating for traditional Chinese medicine and develop a pratical self-powered acupuncture system. This technology enables rapid, routine regulation of human health at any age, which has potential applications in nearly any setting across healthcare platforms alike.

## 1. Introduction

The climate change threats the sustainable environment for human beings as the fossil fuels abused; thence, the novel energy technologies have become the focus of research to relieve the consumption of fossil energy resources. The harness of mechanical motions in the environment including mechanical energy, wave, wind, and rain droplets is an important part of that. Particularly, the triboelectric nanogenerators (TENGs), based on the coupling of triboelectrification and electrostatic induction, have emerged as a rising star with the characteristics of small size, lightweight, and easy manufacture and convert low-frequency movement into electrical energy output [1–4]. However, the TENGs still have the disadvantage with low power density, which stumble its development into commercialization. Researches show that the power density of TENGs can be enhanced by surface micro/nanostructures [5–8] and chemical and physical modification [9–14], but the weakness of that is the surface charge density decays rapidly; also, complexity operation influences the efficiency of enhancing the output performance for TENGs [15–18]. The method of nanoparticles doping [19–24], which mixes high-dielectric nanoparticles (metal nanoparticles) into the triboelectric layer, is a conventional method to enhance the output power of TENG. However, the ameliorative effect of that method is suffered from dispersion of nanoparticles and induces the phenomenon of nanoparticles agglomeration, which will result in charge leakage on the surface of tribo-materials and the output of TENG can be decreased. Nowadays, the surface plasmon resonance effect, which is generated from precious metal nanoparticles, has been widely used in the research area of solar cell, photo-catalysis, photoelectric detection and enhancing photoelectric conversion efficiency [25–30]. While that is rarely used to enhance the output performance of the TENG. Our groups originally proposed that the surface plasmon effect enabled metal-grating which can enhance the output performance of the TENG, but some hot electron-holes may disappear with the adoption of metal-grating and the performance (0.4 mW/cm^2^) [31]. Indeed, there are still exit many practical bumps for realizing the goal for TENGs to grow into commercialization.

Thus, we proposed a local surface plasma boosted TENG (P-TENG) via doping with Ag@SiO_2_ core-shell particles (Ag@SiO_2_-NPs) with the visible light illumination, which can deliver high instantaneous power density over 4.375 mW/cm^2^. Firstly, the high-performance TENG is realized via doping core-shell nanoparticles to improve the dielectric properties of triboelectric film and mitigate surface charge leakage to obtain a higher performance. Then, the surface plasmon effect generated from Ag@SiO_2_ NPs can further boost the output performance of the TENG; especially, the shell can extend the existence of the hot electrons-hole pairs and enhance that electric intensity. Additionally, the 300 LEDs can be lightened with the P-TENG possessing a real estate of 4 cm∗4 cm, and a wireless sensing system also is activated further than dozens of meters. This technology offers a general, easy-to-follow method to boost the output of TENGs, which fills in the gap of the research area of boosting the output of TENG. It is worth mentioned that this uniqueness research associates the TENG with Traditional Chinese Medicine (TCM) acupuncture, and the development electric acupuncture system is applied to modulate the cardiovascular function for human, which broaden the application areas of the high-performance TENG.

## 2. Results

### 2.1. High Performance of P-TENG

**Figure 1:**
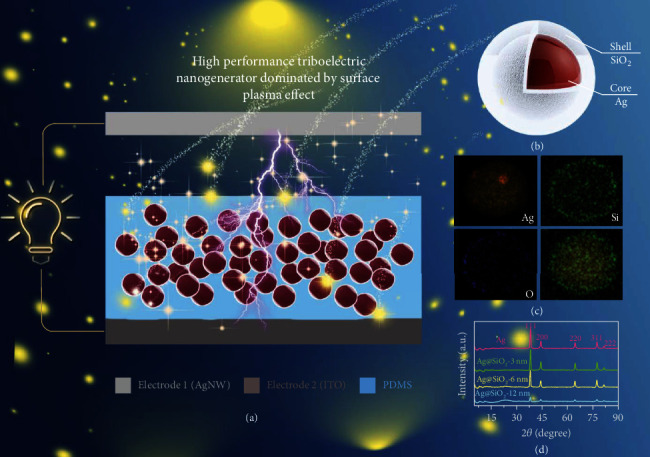
The sketch of the manuscript. (a) The fabrication of the high-performance TENG. (b) The structure of doping nanoparticles. (c) The Energy disperses spectroscopy of the Ag@SiO_2_ NPs. (d) The result for XRD of the Ag, Ag@SiO_2_-3 nm, Ag@SiO_2_-6 nm, and Ag@SiO_2_-12 nm.

The P-TENG fabricates from the basic framework of vertical contact-separation mode, as depicted in Figure 1(a). The P-TENG consists of electrode 1, tribo-surface, and electrode 2 (Ag nanowires, refer as AgNWs; polydimethylsiloxane doping Ag@SiO_2_, refer as PDMS doping Ag@SiO_2_; and indium tin oxide, refer as ITO). The dielectric properties of tribo-material doping with core-shell structure nanoparticles can be boos, the structure and elemental analysis of the core-shell nanoparticles are shown in Figures 1(b)–1(c); thus, the output power of TENG can be enhanced for the first step. Then, the surface plasmon resonance effect is generated via Ag@SiO_2_ with visible light illumination; the output power can be further boosted. The intensity of the surface plasmon effect is directly relevant to the shell thickness, to a certain extent, which means that it can make a difference on the output performance of the TENG. Subsequently, the three shell thickness Ag@SiO_2_ NPs were prepared successfully, and the X-ray diffraction (XRD) results verified that, as depicted in Figure 1(d).

**Figure 2:**
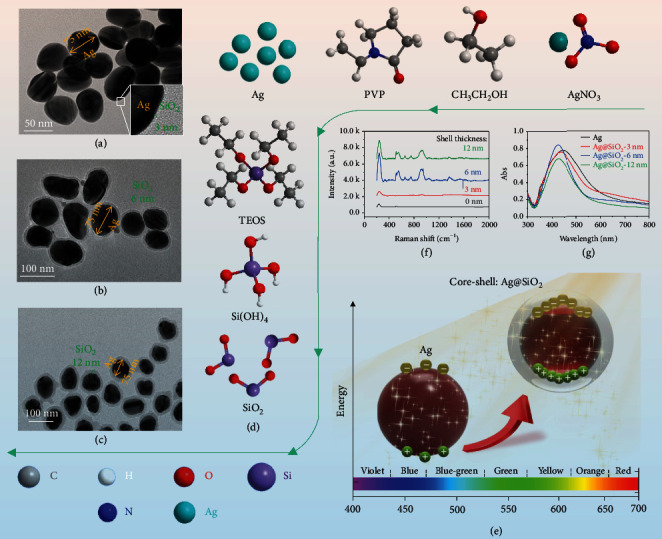
The preparation and characterization of the Ag@SiO_2_ NPs. (a-c) The TEM of Ag@SiO_2_ (shell thickness 3 nm, 6 nm, and 12 nm). (d) The raw materials and intermediate products of fabricating Ag@SiO_2_ NPs. (e) The superiority of Ag@SiO_2_ NPs in generating surface plasmon effect compared with the Ag NPs. (f) The plasmon strength of different shell thickness of Ag@SiO_2_ NPs. (g) The UV-visible absorption spectrum of Ag, Ag@SiO_2_-3 nm, Ag@SiO_2_-6 nm, and Ag@SiO_2_-12 nm.

We discuss the circumstances of doping Ag@SiO_2_ NPs with three shell thickness (3 nm, 6 nm, and 12 nm) in this manuscript; all of them are shown in Figures 2(a)–2(d). It is well known that the different thickness NPs present the different optical properties (Figures 2(f)–2(g)), which can influence the output performance of the TENG. Also, we fabricate a conventional TENG doping with Ag nanoparticles (Ag NPs); results show that the doping core-shell particles have a better dispersion and plasmon enhancement effects (Figure S1 and Figure S2); thus, the higher output performance of TENG will be achieved. And the maximum power reaches the thickness of 6 nm with the visible light (0.6 times solar) illumination. The measured peak output current of P-TENG is about 250 *μ*A, around 10 times higher than that of conventional TENG with PDMS. And the peak output voltage across the load resistor (*R* = 100 *MΩ*) is 1281 V. Moreover, with a wide range of load resistance from 1 k*Ω* to 100 M*Ω*, the maximum output power reaches 70 mW, nearly 35 times higher than the conventional TENG, via the simple way to achieve the target of high-performance TENG.

### 2.2. The Working Mechanism of the P-TENG

**Figure 3:**
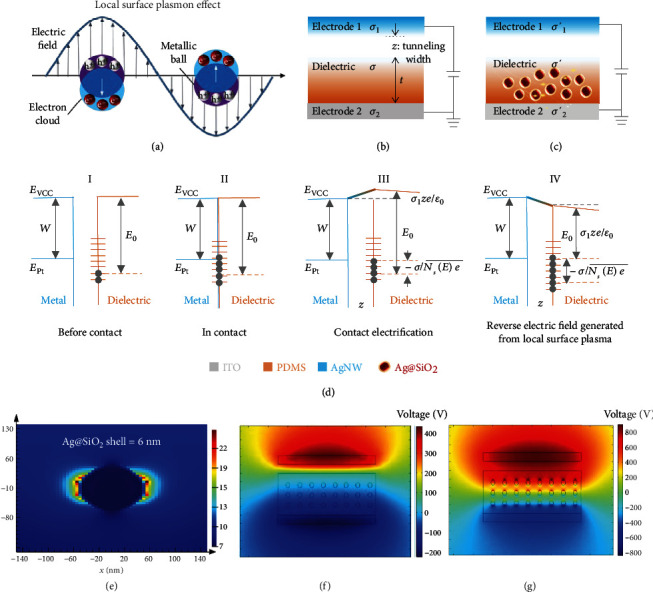
The mechanism of high performance TENG dominated by surface plasmon effect. (a) The mechanism of the local surface plasmon effect generated from metal nanoparticles. (b) The capacitor model of the conventional TENG. (c) The capacitor model of TENG doping with Ag@SiO_2_ enhanced via local surface plasma. (d) Schematic diagram of contact electrification process of the enhanced TENG. (e) The simulation of the surface plasmon effect intensity enabled Ag@SiO_2_ NPs. (f) The simulation for output voltage of doping TENG via COMSOL 5.3a. (g) The simulation for output voltage of the booted TENG via local surface plasmon.

The surface plasmon effect is the consorted with oscillation of free electrons with respect to fixed positive ions in a metal and is one of the most important properties of metals (Figure 3(a)). In most cases, the plasmon frequency of metals is in the ultraviolet region, making the plasmon reflective in the visible range. For general plasmon in the bulk state, plasmon energy *E*_p_ can be represented as follows [32]:

(1) $$\begin{array}{c} E_{p}=h\sqrt{\frac{{ne}^{2}}{m\varepsilon_{0}}}=h\cdot w_{p}, \end{array}$$

where *n* is the electron density, *e* is the electron charge, *m* is the electron mass, *ε*_0_ is the permittivity of the free space, *h* is the Planck constant, and *ω*_p_ is the plasmon excitation frequency. As the metal nanoparticles with the visible light illumination, the local surface plasmon effect can be generated, and the electric field around the NPs which are doped into the tribo-material will be changed.

TENG conforms to the parallel plate capacitor model, an electric field between the electrode 1 and dielectric film will be formed, and which intensity is proportional to the induced charge amounts on the electrode surface. With the effect of the local potential, the charges on the surface of dielectric will reflow to the electrode 1. The output voltage depends on the electric field intensity and the separation distance of the electrode 1 and dielectric. Also, an energy barrier is formed in the separation process to prevent the reflow of electrons. To simplify the model of the TENG generate electricity, a tunnel distance *z* is defined, and if the distance between the electrode 1 and dielectric is less than or equal to *z*, the electrons can transfer from the two surface and maintain a stable Fermi level. For the parallel plate model, the surface charge density of dielectric is *σ*, the induced charge density of electrode1 is *σ*_1_, and the electrode2 is *σ*_2_; the relationship between them should confirm with the following formula:

(2) $$\begin{array}{c} \sigma +\sigma_{1}+\sigma_{2}=0. \end{array}$$

Then, the power densities for TENGs which are doped with composite particles are

(3) $$\begin{array}{c} \sigma^{\prime }+\sigma_{1}^{\prime }+\sigma_{2}^{\prime }=0. \end{array}$$

With the effect of build-in electric field *σ*′/*ε*_0_, the change of vacuum level between metal and dielectric surface changes by Δ*E*_*vcc*_ are

(4) $$\begin{array}{c} \Delta E_{vcc}=\sigma_{1}^{\prime }ze/\varepsilon_{0}, \end{array}$$

where *e* is the elementary charge.

When the system is in equilibrium, the dielectric surface state will be filled up as high as the Fermi energy level in the metal. We assume that the density of surface state is *N*_s_(*E*), the average density of surface state is $\bar{N_{s}\left(E\right)}$, and the range of filled surface state Δ*E*_*s*_ can be described as

(5) $$\begin{array}{c} \sigma =-e\int_{E_{0}}^{E_{0}+\Delta E_{s}}N_{s}\left(E\right)dE, \end{array}$$

(6) $$\begin{array}{c} \bar{N_{s}\left(E\right)}=\frac{\int_{E_{0}}^{E_{0}+\Delta E_{s}}N_{s}\left(E\right)dE}{\Delta E_{s}}, \end{array}$$

(7) $$\begin{array}{c} △E_{s}=-\sigma /\bar{N_{s}\left(E\right)}e. \end{array}$$

From Equation (3) and Equation (6):

(8) $$\begin{array}{c} E_{0}-W=\Delta E_{vcc}+\Delta E_{s}=\sigma_{1}ze/\varepsilon_{0}-\sigma /\bar{N_{s}\left(E\right)}e, \end{array}$$

where the *E*_0_ is the initial energy level range of the dielectric and the *W* is the work function of the metal. And the output voltage of TENG is *V*, and the surface charge density of the dielectric is *σ*, as shown in the following formulas:

(9) $$\begin{array}{c} V=\frac{\sigma_{1}}{\varepsilon_{0}}z-\frac{\sigma_{2}}{{\varepsilon \varepsilon }_{0}}t, \end{array}$$

(10) $$\begin{array}{c} \sigma =\frac{V+\left(\left(W-E_{0}\right)/e\right)\left(1+t/\varepsilon z\right)}{t/{\varepsilon \varepsilon }_{0}+\left(1/N_{s}\left(e\right)e^{2}\right)\left(1+t/\varepsilon z\right)}. \end{array}$$

The output voltage *V* has a negative correlation with the dielectric properties *ε* of the tribo-material. When doping the core-shell Ag@SiO_2_ NPs into the PDMS, the numeric of *σ* will be increased via the analysis from the intrinsic equation, thus the *V* can be boosted and the numeric addition of surface charge density will present for the first. Then, as the doping Ag@SiO_2_ NPs are illuminated with the visible light, the local surface plasmon effect can be generated, and the stimulated free electron gas around precious metal oscillates collectively when coupling with electromagnetic wave; thus, the electric field are forming around the NPs. With the effect of the electrostatic field generated from the dielectric and electrode 2, the direction electric fields are formed around the NPs, and the electric fields generated from NPs can be changed under the electrostatic force which generated from the triboelectric nanogenerator, presenting a vertical state and in opposite to the electrostatic field. From Equation (3), *E*_*vcc*_ increases with the generation of the local surface plasma; the average density of surface state $\bar{N_{s}\left(E\right)}$ and the range of filled energy level Δ*E*_*s*_ are also boosted. Automatically, the output voltage *V* of the TENG and the surface charge density *σ* can be boosted. Theoretical analysis shows that the local surface plasmon effect generated from the Ag@SiO_2_ NPs does boost the output performance of the TENG, as shown in Figures 3(b)–3(d).

And a series of related simulations have been carried out to verify the mechanism of surface plasmon effect via Ag@SiO_2_ NPs for boosting the output performance of the TENGs. The software of FDTD was utilized to simulate the surface plasmon effect generated from Ag NPs and Ag@SiO_2_ NPs. Results prove that the intensity of the surface plasmon effect via Ag@SiO_2_ NPs is indeed over the Ag NPs under the same triggering condition, as depicted in Figures 3(e) and S3(a)–S3(b). Further, the resonance will exit the resonance between two particles at a proper distance, and the intensity of the surface plasmon effect can be further enhanced, as shown in the Supporting Information Figures S3(c)–S3(d). Moreover, the output performance of P-TENG dominated by the surface plasmon effect are also investigated utilized the software of the COMSOL 5.3a; we simulated the doping NPs in the excited stage, which produce an opposite electric field inside of the tribo-materials, and that possess a higher energy level compared with PDMS. Thus, the output voltage reached dozens of times than the TENG only doped with the Ag@SiO_2_ NPs, and the results can validate the efficiency of the surface plasmon effect for enhancing the output performance of the TENG; also, the simulation results are depicted in Figures 3(f)–3(g).

### 2.3. Influence of the Shell Thickness on P-TENG's Output Performance

**Figure 4:**
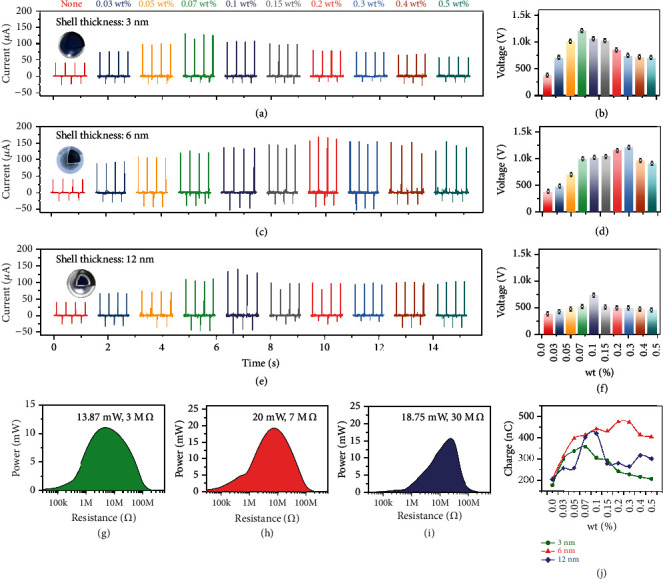
The output performance of the P-TENG doping with Ag@SiO_2_ in the ambient environment. (a-b) The output current and output voltage of Ag@SiO_2_-3 nm. (c-d) The output current and output voltage of Ag@SiO_2_-6 nm. (e-f) The output current and output voltage of Ag@SiO_2_-12 nm. (g-i) The output power of Ag@SiO_2_-3 nm, Ag@SiO_2_-6 nm, and Ag@SiO_2_-12 nm. (j) The charge variation of Ag@SiO_2_-3 nm, Ag@SiO_2_-6 nm, and Ag@SiO_2_-12 nm.

Three shell thickness of the Ag@SiO_2_ (3 nm, 6 nm, and 12 nm) were prepared to investigate the domination of filler silica proportion on the output performance of P-TENG. Mix all the types of Ag@SiO_2_ NPs with the mass fraction of 0%, 0.03 wt%, 0.05 wt%, 0.07 wt%, 0.1 wt%, 0.15 wt%, 0.2 wt%, 0.3 wt%, 0.4 wt%, and 0.5 wt%, and the output performances of the TENG are diagrammed in Figure 4. Figures 4(a)–4(b) portrait the output performance of P-TENG doping with Ag@SiO_2_ NPs with SiO_2_ shell thickness of 3 nm. The output current can be enhanced with the increase of doping content, and the maximum value 131 *μ*A appears at the contents of 0.07 wt%, nearly 4 times higher than the current of the conventional TENG with PDMS (36 *μ*A). Then the current decrease with the doping content increasing, and the current level off at 68 *μ*A, which are higher than the TENG with PDMS, even the TENG with the Ag NPs, thanks to the existence of the modified shell SiO_2_.

The output voltages have the same variable trend as the output currents; the highest output voltage reaches 1.27 kV at the doping content of 0.07 wt% and then drops down to 704 V with the doping content increment, while the voltage of conventional TENG is only 368 V. Figures 4(c)–4(d) diagrammed the output performance of doping Ag@SiO_2_ NPs with shell thickness of 6 nm, and the output currents and voltages are higher than the TENG with 3-nm shell thickness in general. The highest current 172 *μ*A is obtained at the doping content of 0.2 wt%, and with the continued content enhancement of the Ag@SiO_2_ NPs, the output performance drops down slightly. Interestingly, the highest output voltage of shell thickness 6 nm is 1.17 kV, which is lower than the value of 3 nm thickness. Afterwards, the output performance of 12-nm shell thickness was discussed, and the output currents are diminished compared to the value of the 6 nm thickness, but that is higher than the thickness of 3 nm. The highest current was reached 141 *μ*A at doping content of 0.1 wt%, and the output voltage was 739 V.

To emphasize the superiority of the doping shell thickness Ag@SiO_2_ NPs, the output performance of TENG doping Ag NPs was also explored, and the results are shown in the Supporting Information Figure S4. The maximal output current of TENG with Ag NPs is merely 65 *μ*A, and the output voltage is 632 V, mainly due to the agglomeration of the nanoparticles, which lead to the seepage effect and make the charges on the tribo-material leak out quickly. The results turn out that the modified SiO_2_ shell not only can boost the output performance of the TENG due to the high dielectric properties, but also present a barrier that can impede the surface charges leakage. It means that the higher output performance TENG are achieved compared to the conventional doping method.

Furthermore, the output powers have been calculated, for a large range of load impedance from 1 k*Ω* to 100 M*Ω*; the output power of 3-nm shell thickness is 13.87 mW at the load of 3 M*Ω*, 20 mW at the resistor of 7 M*Ω* belongs to 6-nm shell thickness, and the power of 12-nm shell thickness is 18.75 mW with the load of 30 M*Ω*, as diagrammed in Figures 4(g)–4(i). Results shows that the TENG with Ag@SiO_2_ NPs gets the power dozens for the conventional TENG (2.8 mW), which are shown in the Supporting Information Figure S5 and verify the feasibility of our proposal at the first step. In addition, the charges of the P-TENG are counted, the maximum charge is 474 nC, and the changing curve is shown in Figure 4(j).

### 2.4. Direct Observation of the Plasmon Resonance Effect Enhancing Output Performance

We assumed that photoelectric characteristic of the NPs which will change the electric field distribution of the tribo-materials could enhance the output performance of the TENG and did a series of experiments to validate our assumption. The Xenon lamp was chosen as the light source to illuminate Ag@SiO_2_ NPs to generate the local surface plasmon effect; also, the fundamental Ag NPs were first investigated to prove our hypothesis, and the results are shown in the Supplementary Figure S5(a)–S5(d). First on, we put the lamp at the private float angle of 60° with a 0.2 times solar illuminance to radiate the working TENG's tribo-materials doping with Ag NPs. The output currents have an enhancement in contrast with the TENG without light, and the highest output reaches 69 *μ*A at the doping content of 0.07 wt%. Then, we increase the light intensity and observe on the output variation of TENG. The output current gets a further increase, the output current up to 92.5 *μ*A, more than 45 percent than the TENG without light, as shown in the Figure S5c. The output voltages of TENG with Ag NPs are shown in Figure S4(d), and the highest is 735 V, compared to the TENG without light, which gets an dozens enhancement of 103 V. Experimental results show that the output performance has a positive correlation to the intensity of plasmon excitation intensity, if an improving electromagnetic field generated from local plasmon effect enabled NPs, the output performance of TENG can be further boosted.

**Figure 5:**
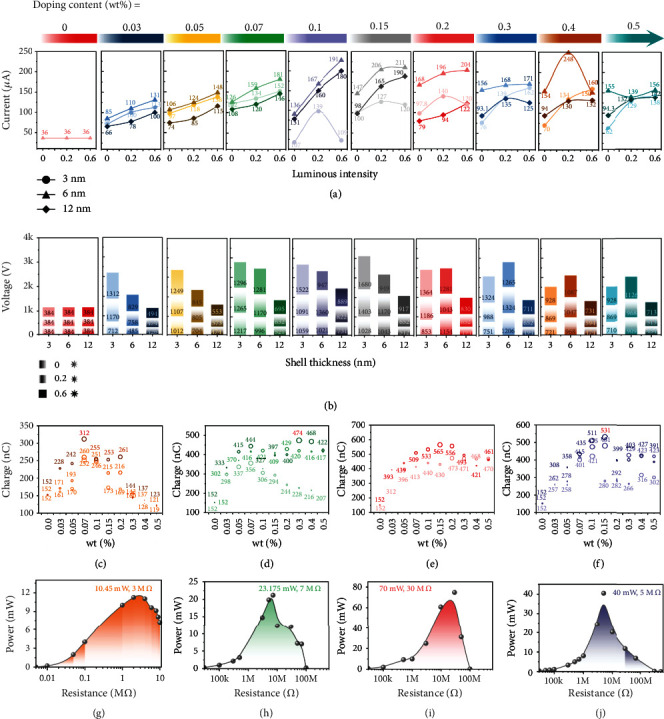
The output performance of P-TENG enhancing via the surface plasmon effect. (a) The output current of Ag@SiO_2_-3 nm, Ag@SiO_2_-6 nm, and Ag@SiO_2_-12 nm with visible light. (b) The output voltage of Ag@SiO_2_-3 nm, Ag@SiO_2_-6 nm, and Ag@SiO_2_-12 nm with visible light. (c-f) The output voltage of Ag, Ag@SiO_2_-3 nm, Ag@SiO_2_-6 nm, and Ag@SiO_2_-12 nm with visible light. (g-j) The output power of Ag, Ag@SiO_2_-3 nm, Ag@SiO_2_-6 nm, and Ag@SiO_2_-12 nm with visible light.

Hence, the P-TENG doping into core-shell Ag@SiO_2_ NPs with light is consecutive to study, and the output performance of the P-TENG is depicted in Figure 5. Figure 5(a) shows the variation curve of the output current at different doping contents, which contains the TENG with light and without light illumination, and three shell thickness Ag@SiO_2_ NPs were compared. The P-TENG with the 3-nm shell thickness acquire the lowest output current in contrast with the other two thickness, and the generated local surface plasmon effect has a little impact on the output even increase the intensity of the illumination light. The output currents of TENG with doping content ranging from 0.03 wt% to 0.07 wt% all have an improvement with the increase of light, while the current drops down with the enhancement of the light intensity due to the surface charge overflow with absorbing the photon energy and increasing less than dissipating. When the doping content continues increase, the currents are enhanced further as the light intensity lifting, and the highest output current of 3-nm thickness is 162 *μ*A at the doping content of 0.3 wt% eventually. The output current of P-TENG with 6-nm shell thickness NPs presents another trend, which takes the current above all the doping materials; also, the best plasmon effect enhancing the output current is realized by this NPs. The highest output current 248 *μ*A was reached at the doping content of 0.4 wt%, which means that this shell thickness and the doping content are suitable for our research experiments. Subsequently, the output current declines as the shell thickness increases to 12 nm, while the intensity of the local surface plasmon effect for boosting output is weakened, and the peak value 190 *μ*A is obtained at the doping content of 0.15 wt%. The regularity of TENG's output is rigidly related to the intensity of the local surface plasmon effect. Figure 5b depicts the output voltage (with the resistance of 100 M*Ω*) of three shell thickness Ag@SiO_2_ NPs; the voltages have the same tendency as the output current and get the highest 1680 V at doping content of 0.15 wt% with the 3-nm shell thickness. Even more interesting is that the output voltage has a close connection with the shell thickness and decreases as the shell thickness increasing. Also, the charges are calculated through the integration from a cycle output current, the maximum is 565 nC, which has the dozen times of the conventional TENG; the results are depicted in Figures 5(c)–5(f). In addition, the peak output powers are also presented via the highest output current with the resistance range from 1 k*Ω* to 100 M*Ω* for each shell thickness, as shown in Figures 5(g)–5(j); the maximum power can reach to 70 mW at the resistance of 30 M*Ω* with device size of 4 cm∗4 cm; the process for TENG's output performance enhancement is presented in the Supporting Information Video S1.

Additionally, the efficiency of the surface plasmon effect for boosting the P-TENG's output performance has been investigated. The doping content 0.15 wt% for three sorts of Ag@SiO_2_ NPs were adopted to verify that, as shown in Figure S6a. The output current reaches nearly 150 *μ*A with visible light, and then setting the P-TENG in the darkness for two hours, the current drops down to the output level of that TENG only doping with Ag@SiO_2_ NPs. When the P-TENG is irradiated with the visible light once more, the output current comes a sharp rise in one minute. Whatever the shell thickness is, the regular phenomenon recurs all the time. In addition, the P-TENGs are also irradiated with various wavelengths, and the output current reduces at the irradiation of UV-light, because the higher energy of the UV-light induces the electrons on the tribo-materials escaped into the air; the results are shown in Figure S6b. The durability of the P-TENG with 6-nm shell thickness (0.1 wt%) is also studied, the output current still maintains a high output performance after hundreds of cycles contact, and the results are depicted in Figure S6c. The P-TENG can power low-power devices such as charging the capacitor (Figure S7a); the capacitances from 10 *μ*F to 100 *μ*F were chosen. The results show that the smaller the capacitance 10 *μ*F of the capacitor is, the shorter charging time is used (6 s) to achieve the target, while the large capacitance (100 *μ*F) of the capacitor possesses the longer charging time used (150 s). Moreover, we successfully light up 300 LEDs leveraging on the high output power of the P-TENG, as shown in Figure S7b and Supporting Information Video S2. Such high output power also allows us to power various electronic devices wirelessly, using a thermometer sensor, a wireless transmitter module, a wireless receiver, and an upper-computer display; the wireless sensor system was contrast; and the sensing data can also be received from more than ten meters. This can be revised as "When the charge capacitor (940 *μ*F) voltage reaches 3 V, the sensor first get the energy and then the wireless trasferring, that's the reason for the twice dropline for the charging curve, as depicted in Figure S7c and Supporting Information Video S3.

### 2.5. Electroacupuncture Dominated by the P-TENG

**Figure 6:**
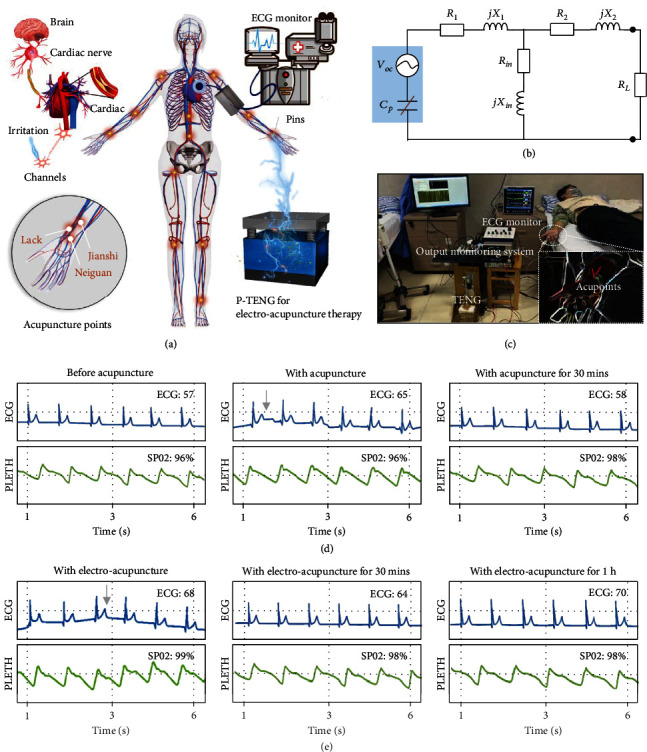
The electroacupuncture dominated by the P-TENG. (a) Stimulation to vague nerve for heart adaptation function via P-TENG. (b) Electrical model of the self-powered electroacupuncture system. (c) Application of the self-powered electroacupuncture system on the healthy human. (d-e) The recorded ECG rate and PLETH value with acupuncture and with electroacupuncture (acupoint of Jianshi and Neiguan).

Cardiovascular disease is one of the most dangerous diseases to human life and often occurs with outdoor recreation or someone in a tense anxiety [33–35]. Acupuncture treatment is a gentle, accessible and relaxing way for the cardiovascular disease, which is highly effective and widely used in clinical treatment on various diseases [36–38]. These days, the traditional acupuncture usually combines with the electrical stimulation to improve the therapeutic efficiency and has a significant curing effect. However, the power supply way is still a block for electric acupuncture therapy development. In consequence, we leverage the high-performance P-TENG to fabricate the self-powered electroacupuncture system for accommodating the function of cardiovascular system, which can be applied in sports first aid, reduction of anxiety, and for aided clinical diagnoses and treatment of some cardiopathies. We utilize the acupoints like Jianshi and Neiguan belongs to heart sutra and the Lack acupoint belongs to lung channels to illustrate the function of the self-powered electric acupuncture based on P-TENG; the process is depicted in Figure 6a; also, the ECG monitor is exploited for displaying the health index during the acupuncture process. The point is that the electric acupuncture generally uses the milliampere-current pulse to stimulate the acupoint; thus, we leverage the matron interface to buck the signal of P-TENG from high voltage low current pulse to a low voltage higher current pulse, which is coincidence with the existed electroacupuncture machines (Figure S8(a)). To express the signal transmission more clearly, the electronic model is shown in Figure 6(b), the simplified equivalent model of P-TENG can be expressed as a voltage source, the transformer equivalent model can transfer into resistances and inductors in parallel, and the variable resistance RL represents skin tissues. As the P-TENG starts to work, the generated electric signals transmit through the buck circuit and convert into required signals, as exhibited in Figure S8(b).

Figures 6(c) and S9 illustrate the self-powered acupuncture system in adjusting the heart function for human in hospital. The physical changes of five healthy volunteers are recorded via the ECG monitor, which documents the electrocardiogram (ECG) and PLETH in normal state, with the acupuncture state and with electric acupuncture state. Figures 6(d) and 6(e) present the physical characteristic of volunteer 1, who is stimulated with acupoints of Neiguan and Jianshi. As shown in Figure 6(d), the ECG is about 57 in the normal station, and the value rises to 65 with acupuncture which also brings heartbeat fluctuation. Then, the physical activity plays a key role, and the ECG of the volunteer 1 begins to level off, which shows the value of 55 with acupuncture after 30 mins; only modest rises compared with the normal state. The next electric acupuncture experiment has been carried out, the ECG value of the body rises rapidly with fluctuation to 68, and the value trends to stable 64 with electrical stimulation after 30 mins. The ECG increases to 70 with the electrical stimulation for 1 h, which indicates that the self-powered acupuncture system has a better control for the health index, but the variation of PLETH is not obvious for the whole experimental process (Video S5). Volunteer 2 was also stimulated with the same acupoint, whose physiological index also varies with external stimulus. But the regulation of the electric acupuncture is a bit weak; the index has a slight variance with the normal condition (Figure S9a). Additionally, the other cardiac channel lack has been utilized to confirm that the system is feasible and stable, as is shown in Figures S9(b)–S9(d). Three-piecer including man and woman were stimulated with Lack and Neiguan acupoint, the physiological index changes when the stimulated condition changes, and all the ECG values are more sensitive to the electronic acupuncture. The above medical experiments were under the guidance of professional doctors, which comply with ethical rules of the hospital of Chongqing University.

## 3. Discussion

In summary, we propose the surface plasmon effect generated from core-shell Ag@SiO_2_ for boosting the output power of TENGs. On one hand, the output performance is enhanced via core-shell Ag@SiO_2_ NPs by the shell can prevent the charge leakage inducing by the metal nanoparticles. On the other hand, utilizing the surface plasmon effect generated by the core-shell Ag@SiO_2_ NPs, the output performance can be enhanced several times than the conventional TENGs. Three sorts of different shell thickness for Ag@SiO_2_ NPs are utilized to explore the influence on the output performance of TENG. And the peak output power of 70 mW is obtained at the shell thickness of 6 nm with the doping content of 0.4 wt%, the output current is 248 *μ*A, and the output voltage is over 1 kV, far beyond the conventional TENG. Benefiting from such high power, we directly light up 300 LEDs and realize a wireless sensing system power off. The P-TENG we proposed has demonstrated an easy method to realize a high performance TENG fabrication. Something to be mentioned is that an application combines the high-performance TENG with the traditional Chinses acupuncture, which broaden the area of the application for TENGs.

## 4. Materials and Methods

### 4.1. The Materials of Core-Shell Ag@SiO_2_

The Ag@SiO_2_ composite nanoparticles with a core-shell structure were prepared in remarkably simple chemical reduction ways. And the preparation process was composed of two parts. One was the Ag nano-sphere preparation and silica-shell coating; the Ag can be obtained through the materials 0.1 g PVP, 1 mL Ag@SiO_2_ (0.1 mol/L), and 100 mL CH_3_CH_2_OH; these materials should be ultrasonic mixing evenly in one conical flask. Then, heating the mixed solution in water bath at 80 °C for 60 min and the Ag NPs were obtained. Next, the Ag@SiO2 NPs were prepared; the materials of 20 mL Ag colloid, 37.5 mL CH_3_CH_2_OH, 12.5 mL pure water, and 2.5 mL ammonium hydroxide were mixed through the ultrasonic agitation for 30 mins. Then, the TEOS was added slowly to mixed solution, which was agitated in the dark for some time, and time direct influences the shell thickness of the Ag@SiO_2_ NPs; the fabrication process of Ag@SiO_2_ NPs is shown in Supporting Information Figure S10 and Table S1.

### 4.2. Fabrication of P-TENG

The P-TENG contains an electrode one (AgNWs), a triboelectric film of PDMS with core-shell Ag@SiO_2_ NPs, an electrode two (ITO), and the triboelectric film spin through the glue homogenizer (EasyCoater 4, Schwan) on the ITO electrode. The PDMS films were prepared from Sylgard 184; it contains two parts (group A and group B). The group A doping with NPs was mixed thoroughly via deaeration mixer (THINKY MIXER AR-100) for 5 mins and then added the group B into the mixer and continue stirring for 5mins, and another 2 mins were used to be defoaming. Then, the obtained mixture was spun on the ITO via 350 rpm which was generated by the spinner, and back dry at the temperature of 75 °C. A P-TENG with the size of 4 cm∗4 cm was fixed at the linear motor (TS-40GZ495-32, Frequency 2 Hz, Force of 40 N) for measuring the output electricity.

### 4.3. Fabrication of the Lighting System

The lamp provides light radiation (CEL-HXF300-T3), and the lamp was put onto the lifting platform (LFP200) and sets as a 60° radiation source. In addition, a filter (UVIRCUT400) was fixed on the lamp for providing a visible light illumination to boost the output performance of TENG.

### 4.4. Characterization

The output currents were measured by a current preamplifier (SR570, Stanford Research System), and the output voltages were measured through a digital storage oscilloscope (R&S RTO2064) with a high-voltage probe (Tektronix P6015A, 100 M*Ω*). The output powers are calculated through the formula *P* = *I*^2^_load_∗*R*; the charge *Q* was obtained by making an integration of one cycle current, where *t*_0_ is the time of the beginning of each cycle.

### 4.5. High Output Performance Demonstration

The module of 300 LEDs we utilized to demonstrate the high-power output of the P-TENG with dropout voltage 2 V. For wireless sensing system, the capacitor (940 *μ*F) was used for powering the transmitter module of ZigBee (LRF215C) and a temperature sensor; the receiver module (LRF215U_PA) was powered over its USB connection, and the temperature displayed at the PC system.

### 4.6. Protocols for Human Subject Studies

The study protocol was reviewed and approved by the ethical review committees of Chongqing University Hospital. For the electric acupuncture experiments, the doctor Ouyang operated the acupuncture sessions, and self-powered electric stimulations were under his supervision. The electric stimulation was supported from TENG with same excitation with former test. Transformer turns ratio is 660 : 40, and wire diameters, respectively, are 0.06 mm and 0.41 mm. All the volunteers were fully informed, and they signed a written informed consent prior to the start of the study.

## Data Availability

A data availability statement is compulsory for all research articles.

## Supplementary Materials

Supplementary Materials Supplementary materials are all available. Figure S1: The fabrication of P-TENG. Figure S2: The Ag NPs doping into the PDMS. Figure S3: The simulation of the surface plasmon effect from Ag and Ag@SiO2. Figure S4: The output performance of TENG doping Ag. Figure S5: The output power of conventional TENG. Figure S6: Study on improving the output performance of TENG by surface plasmon resonance. Figure S7: The output performance demonstration for P-TENG. Figure S8: The stimulation from electroacupuncture instruments and TENG with transformer. Figure S9: The record of the volunteers for electric acupuncture. Figure S10: The fabrication process of the Ag@SiO2 nanoparticles. Table S1: The optimized reaction conditions of shell thickness. Video S1: The surface plasma effect is utilized to boost the output performance of the TENG. Video S2: 300 LEDs are lighted via the high performance TENG. Video S3: A self-powered wireless sensing system is realized. Video S4: Doctor operates the process of acupuncture. Video S5: The electroacupuncture dominated by the P-TENG.
