# Supplementary material for: Surface Plasmon Effect Dominated High-Performance Triboelectric Nanogenerator for Traditional Chinese Medicine Acupuncture
Source: Research (Wash D C). 2022 Oct 7;2022:9765634. doi: 10.34133/2022/9765634 (PMC9575470; doi:10.34133/2022/9765634)
Supplement: Supplementary Materials — are all available. Figure S1: The fabrication of P-TENG. Figure S2: The Ag NPs doping into the PDMS. Figure S3: The simulation of the surface plasmon effect from Ag and Ag@SiO2. Figure S4: The output performance of TENG doping Ag. Figure S5: The output power of conventional TENG. Figure S6: Study on improving the output performance of TENG by surface plasmon resonance. Figure S7: The output performance demonstration for P-TENG. Figure S8: The stimulation from electroacupuncture instruments and TENG with transformer. Figure S9: The record of the volunteers for electric acupuncture. Figure S10: The fabrication process of the Ag@SiO2 nanoparticles. Table S1: The optimized reaction conditions of shell thickness. Video S1: The surface plasma effect is utilized to boost the output performance of the TENG. Video S2: 300 LEDs are lighted via the high performance TENG. Video S3: A self-powered wireless sensing system is realized. Video S4: Doctor operates the process of acupuncture. Video S5: The electroacupuncture dominated by the P-TENG. [file 9765634.f1.zip › Supplementary Materials.docx]

**Supplementary Materials**


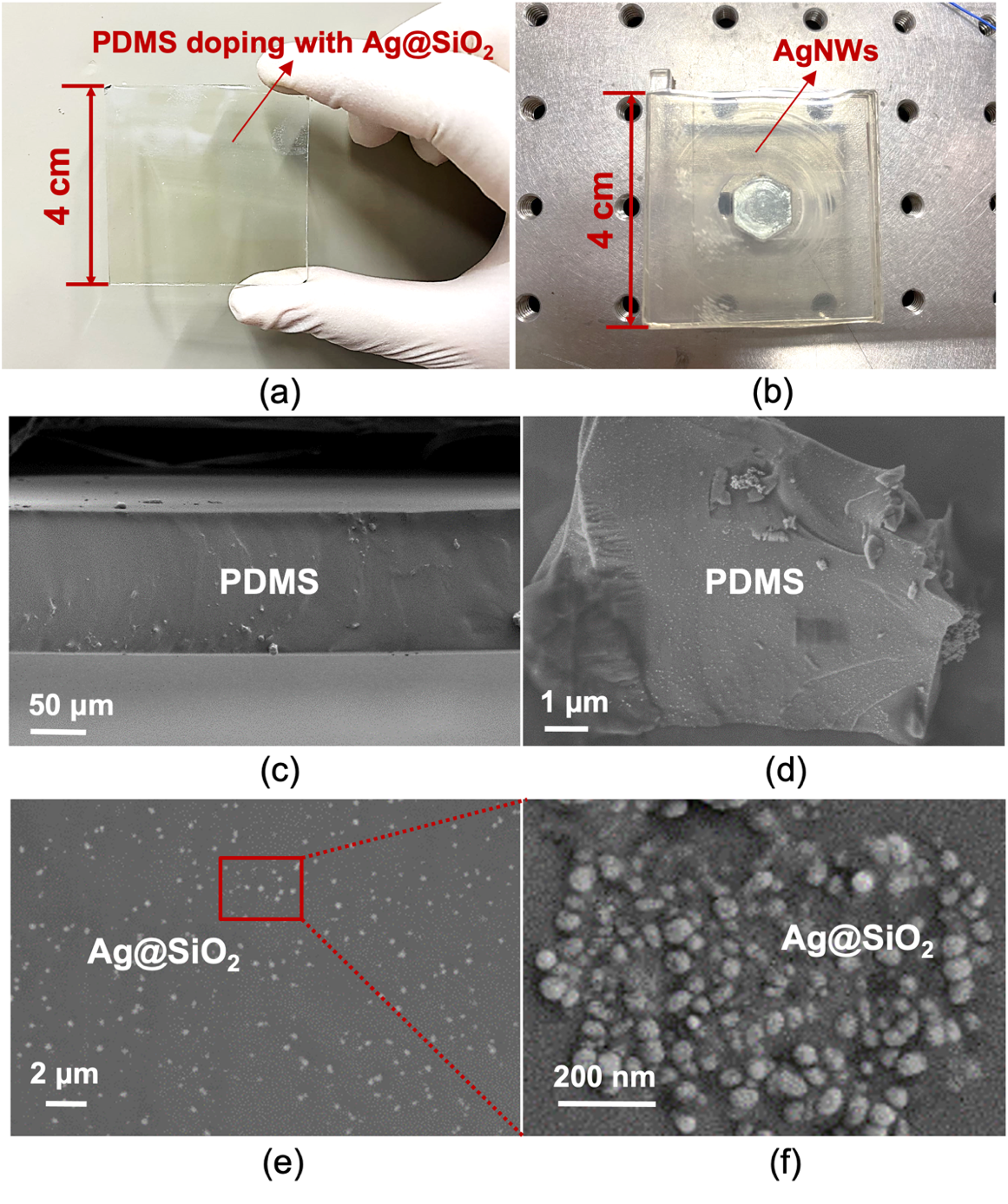


**FIGURE S1.** The fabrication of P-TENG. (a) The tribo-materials PDMS doping with Ag@SiO_2_ spinning on the ITO glass. (b) The Ag NWs electrode is paste on the acrylic. (c) The vertical section of the PDMS film. (d) The cross section of the PDMS film. (e-f) The vertical section of the PDMS film in enlarged size.


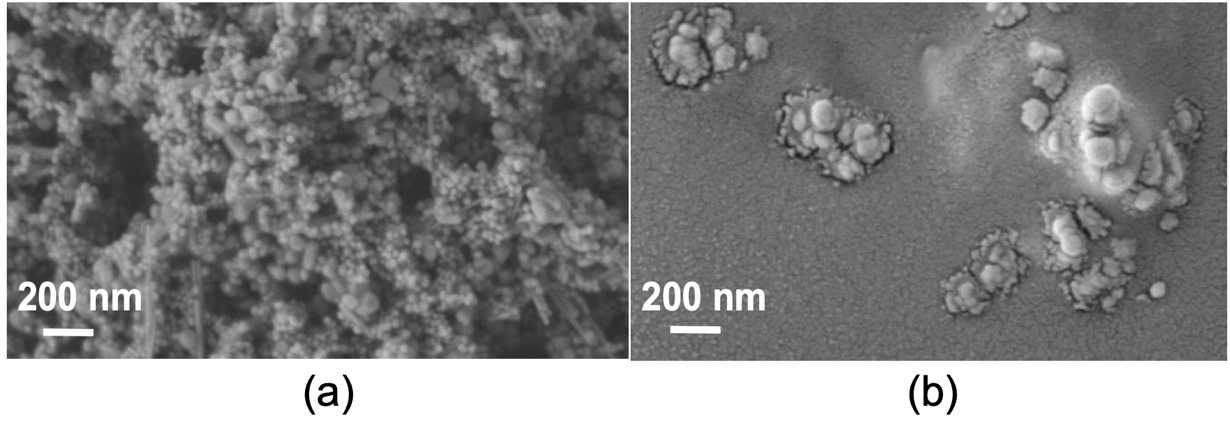


**FIGURE S2.** The Ag NPs doping into the PDMS. (a) The SEM of the Ag NPs. (b) The SEM of the PDMS doping with Ag NPs.


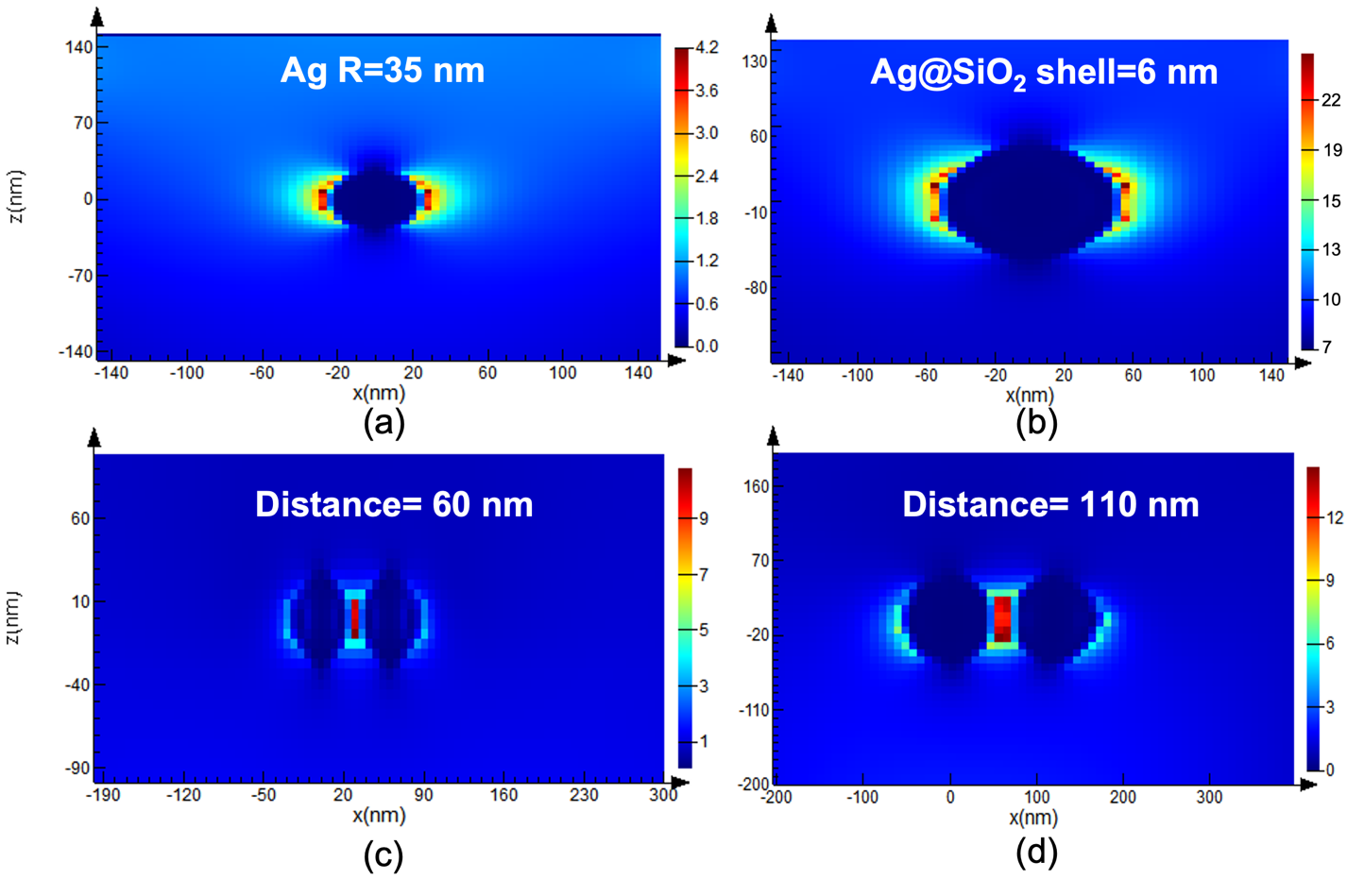


**FIGURE S3.** The simulation of the surface plasmon effect from Ag and Ag@SiO_2_. (a) The intensity of surface plasmon effect generated from Ag. (b) from Ag@SiO_2_ (c) from two Ag@SiO_2_ NPs with a distance 60 nm. (d) from two Ag@SiO_2_ NPs with a distance 110 nm.


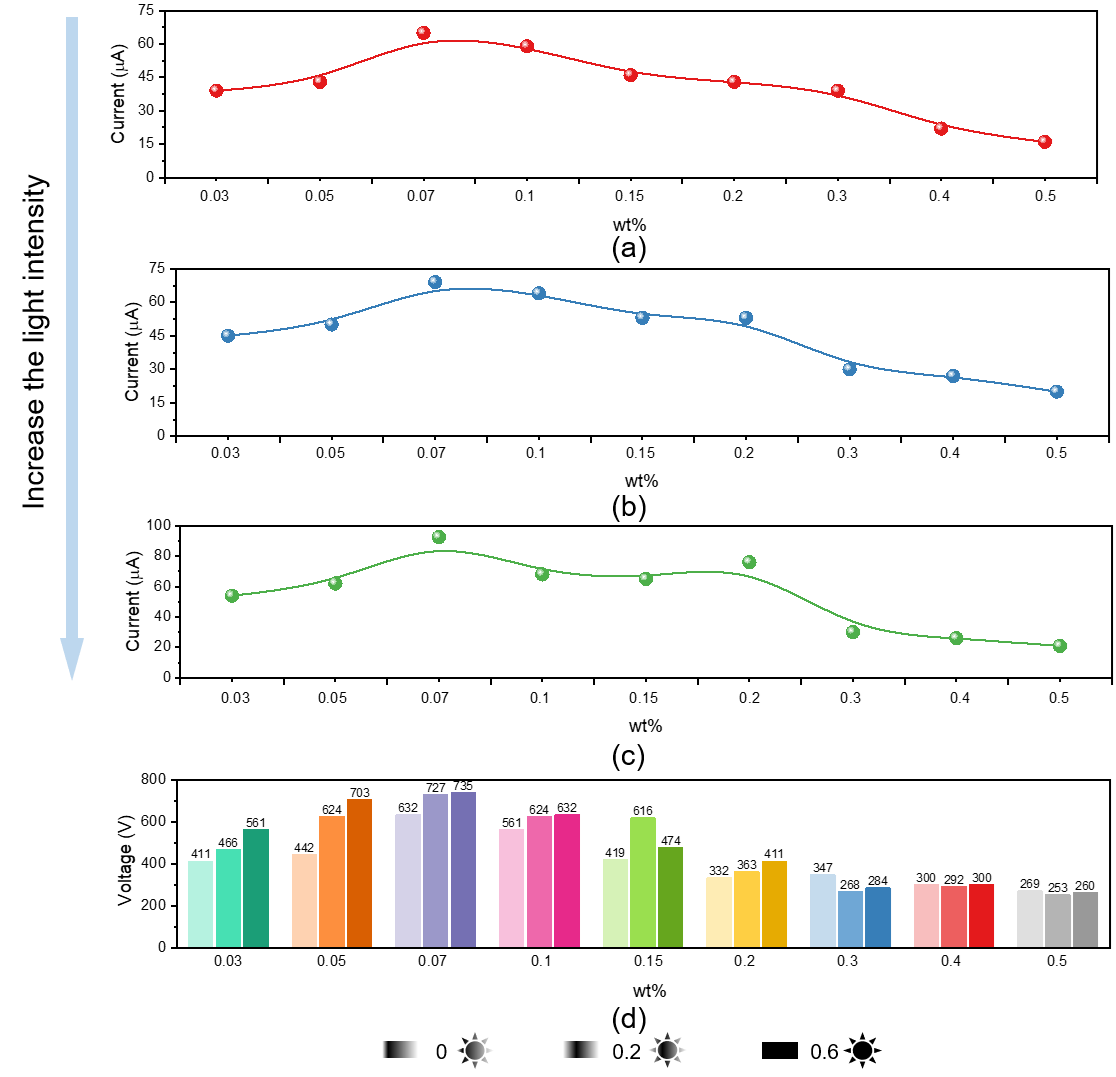


**FIGURE S4.** The output performance of TENG doping Ag. (a) The output current of TENG without visible light. (b) The output current of TENG with 0.2 times solar illuminance. (c) The output current of TENG with 0.6 times solar illuminance. (d) The output voltage of TENG doping with Ag.

**FIGURE S5.** The output power of conventional TENG.


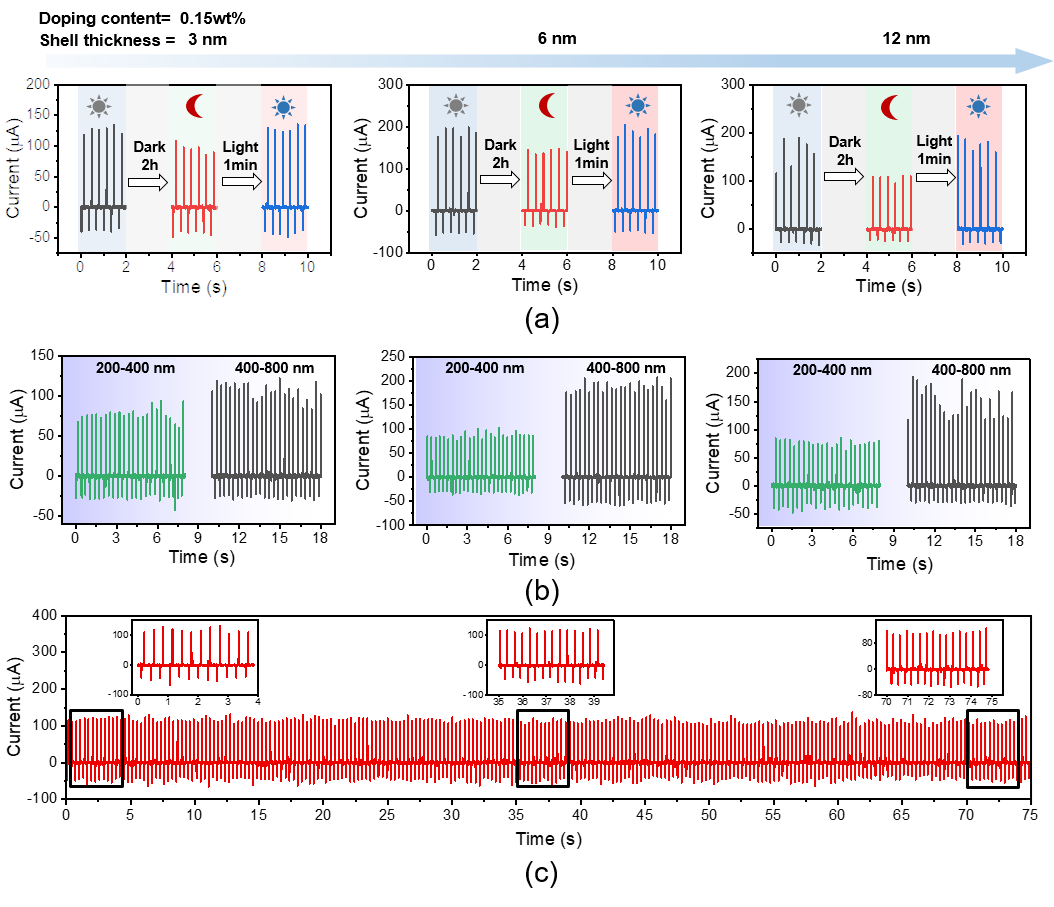


**FIGURE S6.** Study on improving the output performance of TENG by surface plasmon resonance. (a) The effective of surface plasma effect enhancing the output performance of P-TENG. (b) The influence of different band irradiation on the output performance of TENG. (c) The durability of the high performance TENG.


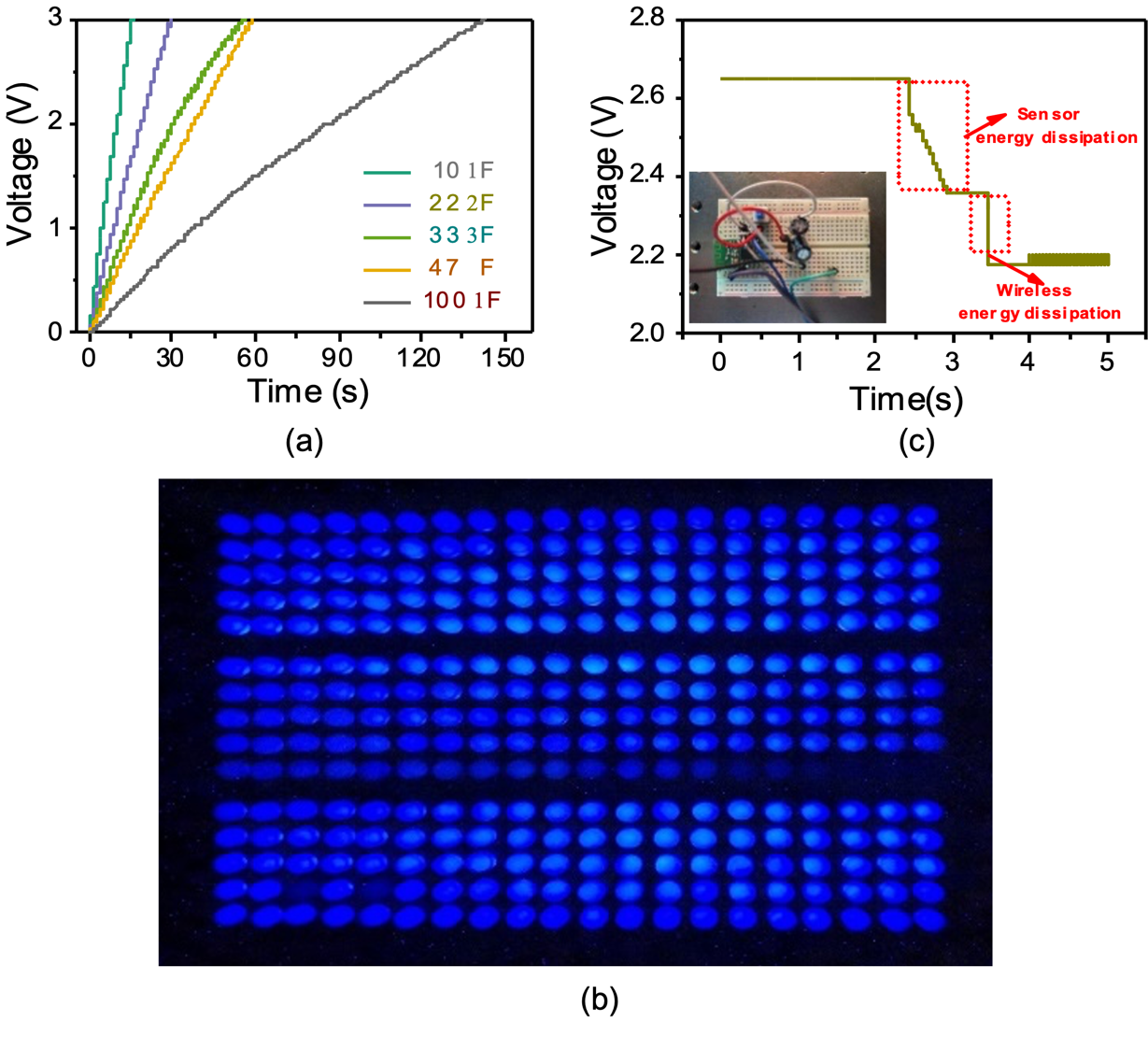


**FIGURE S7.** The output performance demonstration for P-TENG. (a) The charge curve of the capacitor for 10 μF, 22 μF, 33 μF, 47 μF, 100 μF. (b) The 300 leds are lighted up. (c) The self-powered wireless system is realized.


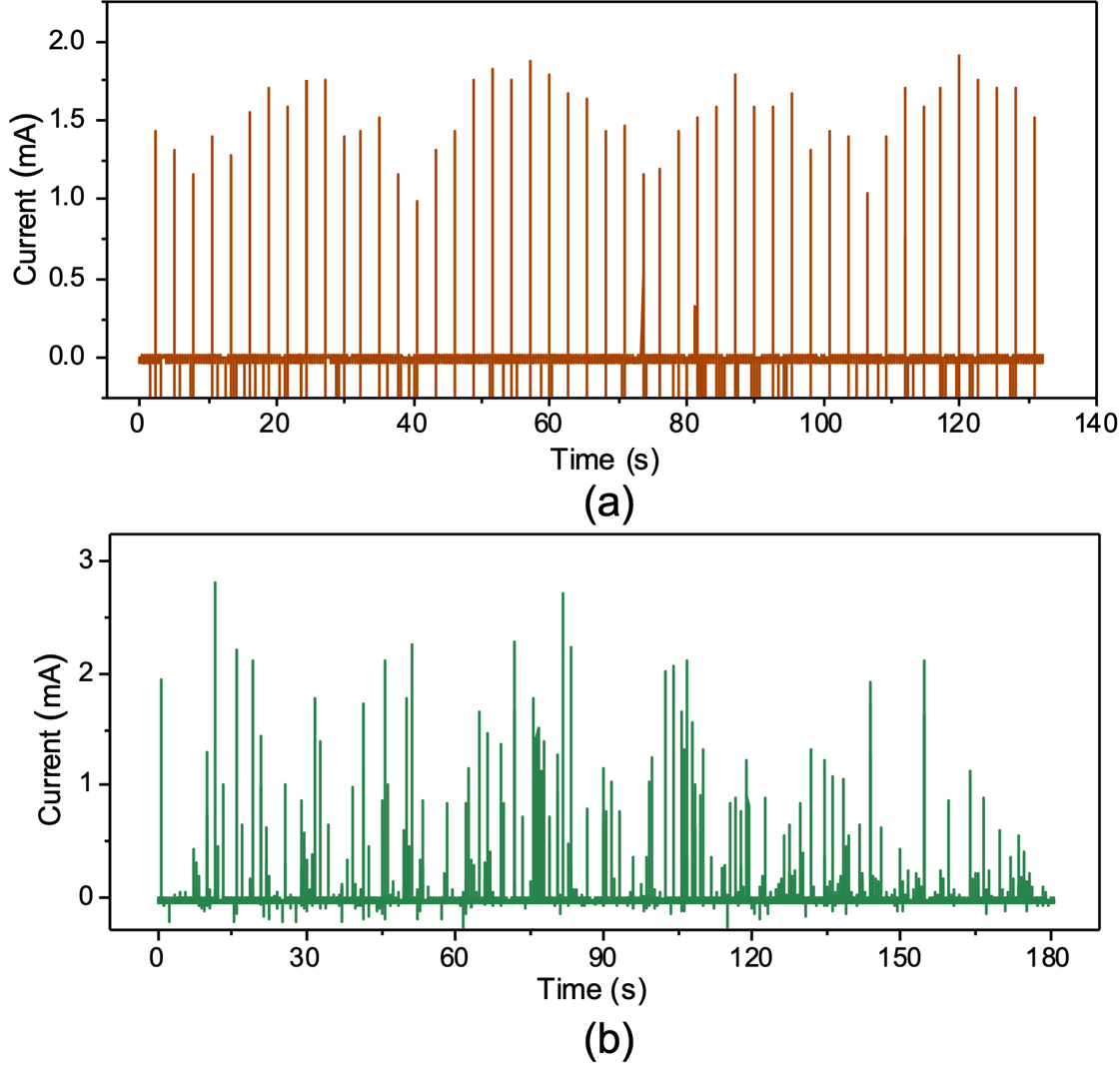


**FIGURE S8**. The stimulation from electro-acupuncture instruments and TENG with transformer. (a) From electro-acupuncture instruments. (b) From TENG with transformer.


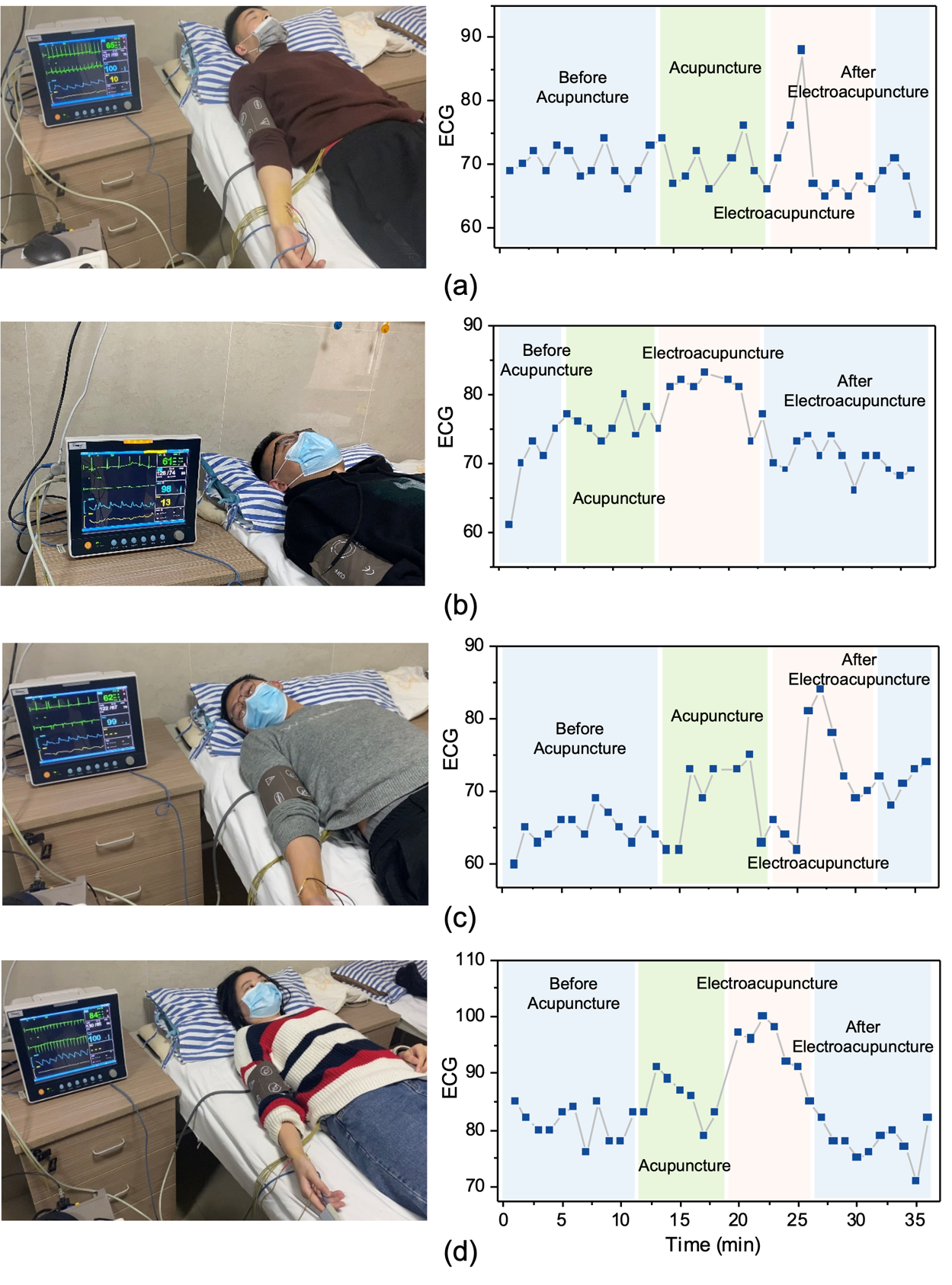


**FIGURE S9.** The record of the volunteers for electric acupuncture. (a) Volunteer 1 and was stimulated with Jianshi and Neiguan acupoints. (b-c) Volunteer 2 and volunteers 3 were stimulated with Jianshi and Lack acupoints. (d) Volunteer 4 (women) was stimulated with Jianshi and Lack acupoints.


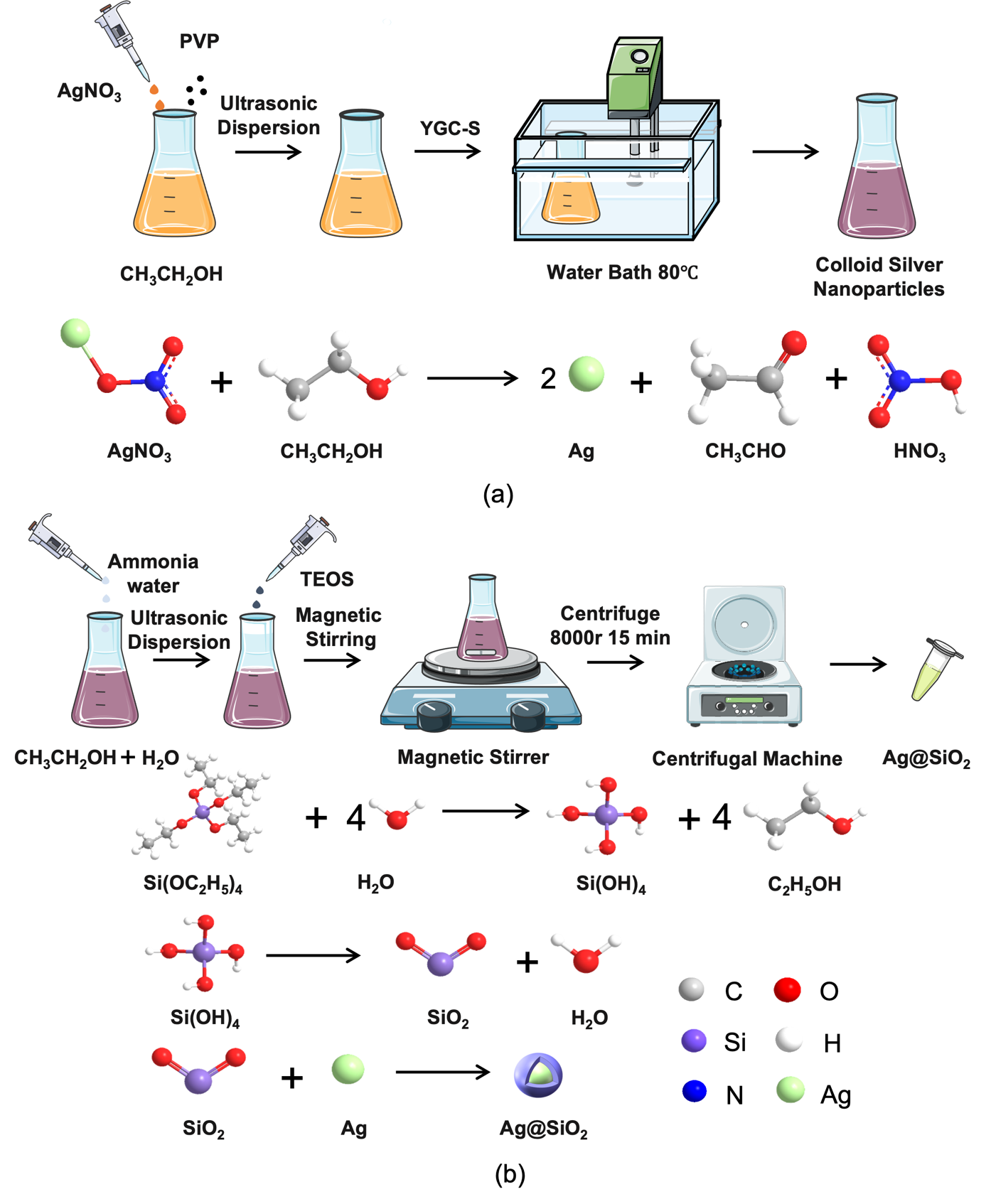


**FIGURE S10.** The fabrication process of the Ag@SiO_2_ nanoparticles. (a) The preparation of the Ag NPs. (b) The preparation of the Ag@SiO_2_ nanoparticles.

The Ag NPs is prepared via the chemical reduction select 0.1 g PVP, 1 ml AgNO_3_ (0.1mol/L) and 100 ml CH_3_CH_2_OH to ultrasonically mix. After the substances were uniformly mixed, putting the conical flask into the water bath pot, heat it to 80 ℃, and keep it at this temperature for 60 min to obtain the Ag NPs.

The Ag@SiO_2_ material is prepared via the Stöber method: select 20 ml Ag colloid, 37.5 ml CH_3_CH_2_OH, 12.5 ml pure water, 2.5 ml ammonium hydroxide to ultrasonically mix. Then a dispersion liquid was formed via ultra-sounding for 30 minutes, passed TEOS into the suspension continuously, and stir in the dark for a period. The Ag@SiO_2_ NPs was obtained.

**TABLE S1.** The optimized reaction conditions of shell thickness.

| The shell thickness | Reaction Time | The capacity of TEOS |
| --- | --- | --- |
| 3 nm | 30 min | 5 |
| 6 nm | 3 h | 5 |
| 12 nm | 3 h | 10 |
